# Chloroform in Dysmenorrhœa

**Published:** 1849-02

**Authors:** J. V. Withers

**Affiliations:** Westpoint, Kentucky


Art. IV.—*Chloroform in Dysmenorrhœa.* By J. V. Withers, M. D., of Westpoint, Kentucky.

I had seen Dysmenorrhœa in my practice, and have carefully perused the history of such cases as are laid down in standard works on diseases of females; yet from all I had seen and read on the subject, my mind was not prepared to believe that the disease could assume so aggravated a form as it did in the case which I am about to relate. I knew it was one of a painful character, but supposed a fatal termination would be rare, if it ever occurred; but the case in hand convinced otherwise; for I

had great reason to apprehend a fatal termination, and that speedily.

I was called to see the case at 8 o'clock, P.M., Nov. 14, The subject of it, a negro girl, aged 20 years, had been taken about an hour previously, up to which time she had enjoyed pretty good health. She represented her menses as having been on her for a day or two; she was suffering with severe bearing-down pains, with general spasmodic action of all the muscles. I examined carefully, and found there was nothing in the uterus to give rise to such contractions and suffering. It was plain that the case was one of dysmenorrhœa, neuralgic in its character. I gave immediately two grains of solid opium, and, after an interval of an hour, twenty drops of laudanum. The opiates seemed to produce no impression upon her, and in thirty minutes I ordered an enema of sixty drops of laudanum; in half an hour I gave twenty drops more, and in half an hour repeated the dose. It was now about midnight, and I had given an equivalent to five or six grains of opium, and no impression had yet been made upon the pain and spasm. I left her, and did not see her again till 11 o'clock next morning. The family told me she remained as I had left her until 4 o'clock in the morning, when she became somewhat easier; she continues so up to this time. She is, however, still suffering. I now ordered two pills of opium, two grains each, one to be given at 1 o'clock, P. M., and the other at 4 o'clock, which was done. At 8 o'clock I again saw her. She was suffering as much as she was the night previous, and the pain was without any intermission; I ordered an injection of sixty drops of laudanum, and also gave thirty drops. All this seemed to produce no impression upon the system, and I requested Dr. Greenley to see her with me. He arrived about 10 o'clock at night. Camphor was combined with the opium, and energetic doses were given repeatedly. At 12 o'clock it was apparent that her sufferings were becoming more acute. In order to overcome the pain and contractions, as opiates had failed, we determined to resort to bloodletting. Blood was accordingly taken from the arm, but without the least benefit. At 3 o'clock we gave her eighty drops of laudanum, and ordered camphor and opium to be given at intervals of an hour.

16th—9 o'clock, A.M. We saw our patient again and found her sufferings on the increase. We now thought it imprudent to push opiates any farther. She had taken an amount equivalent to about thirty grains, without any good result. Again we opened a vein and took blood; put her into a warm bath; produced free purgation by castor oil and spirits of turpentine; but her condition was in no degree bettered, and, as a dernier resort, we applied to chloroform. This was administered at 11 o'clock by means of a pocket handkerchief—the patient was soon brought completely under its influence; fell into a profound sleep; complete cessation of pain is evident; her skin relaxed and moist. I saw her again at 2 o'clock, P.M., and learned that she remained quiet for two hours, experiencing some pain but not sufficient to demand the chloroform. At 5 o'clock I again saw her, and administered chloroform a second time. The effects were instantaneous and perfect. She remained easy until midnight. The pains and muscular contractions then became severe, and I repeated the chloroform. She became easy, and remained so until 4 o'clock in the morning, when restlessness returning I again administered chloroform.

17th. I saw her at 9 o'clock this morning and found her complaining of severe pain. I once more repeated the chloroform, and as in all the other applications it did not fail to give perfect and instantaneous relief. I saw her at 1 o'clock, P.M., and repeated the anæsthetic. She remained easy until 11 o'clock at night, when quite a violent paroxysm came on. At its hight I gave chloroform; the relief was instantaneous. Patient rested easy until 9 o'clock of the night of the 18th, when I again administered the vapor.

19th—8 o'clock, A.M. Chloroform was once more administered.

20th. Patient continued easy up to 7 o'clock, A.M., when I again gave the anæsthetic.

21st. Patient relieved. I depended altogether upon the chloroform for four days in succession. To what extent it may be looked upon as a remedial agent, this case will somewhat illustrate, as well as what claims it may have over opium and bloodletting in subduing pain and spasm. I have detailed all the particulars of this case in order that the comparative virtues of the three might be judged of.

The chloroform was given during a period of nearly eighty hours, varying in the intervals of its administration from four to twelve and twenty hours—its effects lasting the same length of time. The quantity required to produce anæsthesia varied from ten to forty drops, the quantity necessary growing less from the beginning. I saw no ill effects from its use whatever.

Dec. 26, 1848.

